# A High-Resolution Electric Current Sensor Employing a Piezoelectric Drum Transducer

**DOI:** 10.3390/mi12101166

**Published:** 2021-09-28

**Authors:** Wei He

**Affiliations:** School of Information Engineering, Baise University, Baise 533000, China; weiheky@yeah.net

**Keywords:** high-resolution sensor, piezoelectric drum transducer, current sensing, magnetic circuit, electric power grid

## Abstract

A high-resolution sensor using a piezoelectric drum transducer is proposed for power frequency current sensing (50 Hz or 60 Hz). The utilization of the magnetic circuit helps to enhance the response to the electric currents in the power cords. The high sensitivity of the sensor originates from the superposition of the Ampere forces and the amplified piezoelectric effect of the drum transducer. The feasibility of the sensor was verified by experiments. The device exhibits a broad 3 dB bandwidth of 67.4 Hz without an additional magnetic field bias. The average sensitivity is 31.34 mV/A with a high linearity of 0.49%, and the resolution of the sensor attains 0.02 A. The resolution is much higher than that of the previous piezoelectric heterostructure for two-wire power-cords. Error analysis shows that the uncertainty reaches 0.01865 mV at the current of 2.5 A. Meanwhile, the device can generate a load power of 447.9 nW with an optimal load resistance of 55 KΩ at 10A (*f* = 50 Hz) in energy harvesting experiments. The features of high sensitivity, excellent linearity, high resolution, low costs, and convenient installation demonstrate the application prospect of the proposed device for measuring power frequency currents in electric power grids.

## 1. Introduction

Electricity measurement is of great importance for the safety protection, reliability, and early warning of the electrical equipment in electric power grids. The traditional current sensors, such as Rogowski coils [1], Hall devices [2], and current transformers [3], are used for electricity measurement, but there are some disadvantages. For example, Rogowski coils are not suited for small current measurements. Hall devices put forward high requirements for signal conditioners. Current transformers have the shortcoming of signal distortion due to the magnetic saturation. For the measurement of the power frequency (e.g., 50 Hz in China and 60 Hz in North America) currents, a current sensor was developed to use the combination of a piezoelectric cantilever beam and NdFeB magnets [4], which resonates at 60 Hz in operation. However, it is hard to maintain the resonance due to the nonlinear behavior of the device, and a non-resonant device might be preferred in realistic current sensing.

In recent years, magnetoelectric (ME) mechanisms have been reported for energy harvesting [5,6,7,8,9] and current sensing [10,11,12]. ME structures can be used as electric current sensors by coupling the magnetic field generated by the alternating currents [13,14,15,16,17,18]. An ME device with a tunable bias magnetic circuit was presented [13], which exhibits an excellent linearity for 50 Hz current measurement. A ring-type ME structure operating in vortex magnetic field of the currents was developed [14]. The sensor shows a non-resonant sensitivity of ~12.6 mV/A over the frequency range of 1 Hz–30 kHz. A new ME structure was put forward for alternating current sensing [15]. A current sensitivity of 1.03 mV/mA is attained when the input current ranges from 15 mA to 2.1 A. A gradient-type ME current sensor is proposed, which operates in magnetic field gradient detection [16], and a sensitivity of 0.65–12.55 mV/A is attained in a wide frequency range of 10 Hz–170 kHz. ME cantilever beams have also attracted attention due to their high resonant sensitivities. A FeCuNbSiB/Ni/PZT beam was fabricated for coupling the magnetic field around a current-carrying wire with a strong zero-biased response [17]. The resonant sensitivity reaches ~330 mV/A for measuring 50 Hz currents. A current sensor consisting of a FIEA beam, a PZT8 plate, and a permalloy yoke was proposed [18]. The permalloy yoke concentrates the vortex magnetic field of the electric wire to the FIEA beam, which improves the sensitivity of the device. The sensor exhibits a high resonant sensitivity of 300.5 mV/A with a separating distance of 6 mm. However, most of the ME current sensors require DC bias magnetic fields, as the ME effect is strongly dependent on the bias. Furthermore, the presence of the bias magnetic field will increase the volume of the device, which may render its applications impractical.

To measuring 50 Hz or 60 Hz currents in electric power grids, a structure without magnetic field bias might be preferred. A silicon-based piezoelectric current sensing device without a DC magnetic field bias was developed [19]. The device couples the alternating magnetic field of the current-carrying wire and vibrates at the frequency of the current. Recently, current sensors based on piezoelectric cymbal structures have been explored [20,21]. The resolution is 0.05 A [20] and 0.04 A [21] for a single wire and a two-wire power cord, respectively. To improve the resolution, a current sensor using a piezoelectric drum transducer is designed in this paper. The high non-resonant sensitivity results from the superstition of the Ampere forces and the large effective piezoelectric coefficient of the drum transducer. A prototype was fabricated and tested. The results demonstrate the feasibility of the proposed device with high sensitivity and resolution for 50 Hz or 60 Hz current sensing.

## 2. Design and Analysis

**Figure 1:**
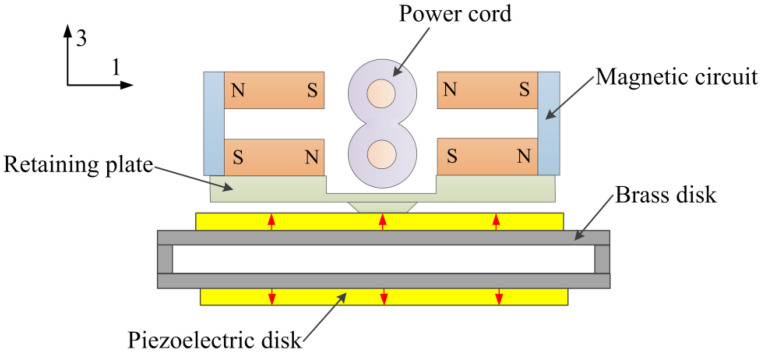
Schematic diagram of the current sensor.

**Figure 2:**
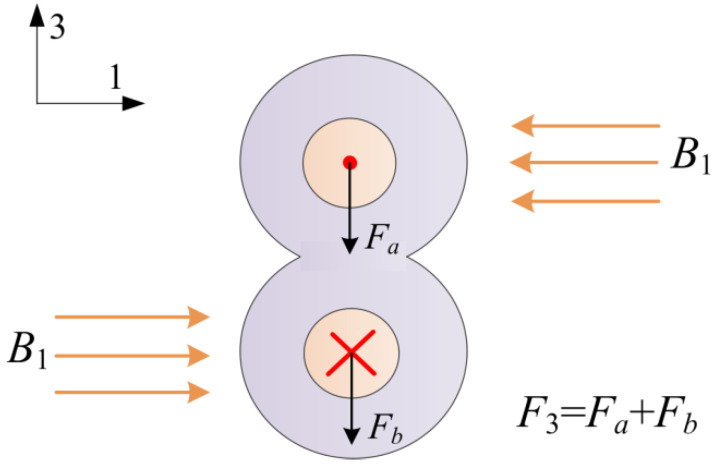
Superimposition of the Ampere forces on the two conductors of the power cord.

Figure 1 shows the schematic diagram of the proposed current sensor. The sensor is composed of a magnetic circuit, a piezoelectric drum transducer, and a mass load. The magnetic circuit consists of four magnets and two magnetic yokes, and the magnetic poles are shown in Figure 1. The drum transducer is made up of a steel ring sandwiched between two composite disks, and the composite disk is constructed from a piezoelectric disk bonded on a brass plate. The magnetic circuit produces magnetic flux density on the conductors of the power cord. When the power cord is energized, the Ampere forces (in 3-direction) on the conductors are superimposed based on Ampere’s force law, as shown in Figure 2. An enhanced reacting force is then induced on a magnetic circuit, and a voltage proportional to the electric current is produced due to the piezoelectric effect of the piezoelectric disks.

As illustrated in Figure 2, the vertical Ampere forces on the two current-carrying conductors (conductor *a* and conductor *b*) can be calculated by

(1) $$F_{a}=J_{0}sin\left(\omega t+\psi \right){∭}_{V_{1}}B_{1}dV\;$$

(2) $$F_{b}=-J_{0}sin\left(\omega t+\psi \right){∭}_{V_{2}}B_{1}dV\;$$

where *J*_0_, ω, and *ψ* respectively denote the current density, angular frequency, and phase angle, $J_{0}=I_{0}/\left(\pi r_{c}^{2}\right)$, *I*_0_ is the amplitude of the current, *r_c_* is the radius of each conductor, *V*_1_ and *V*_2_ are the considered volumes of the conductor *a* and conductor *b*, respectively, and *B*_1_ is the magnetic flux density in 1-direction. The vertical force on the power cord can be expressed as

(3) $$\begin{array}{l} F_{3}=F_{a}+F_{b}=J_{0}sin\left(\omega t+\psi \right)\left(B_{ma}-B_{mb}\right)\;\\ =J_{0}B_{l}sin\left(\omega t+\psi \right) \end{array}$$

where *B_ma_* and *B_mb_* are the integrals of *B*_1_ over the volume *V*_1_ and *V*_2_, respectively.

The reaction force of *F*_3_ (*F_r_*) acts on the magnetic circuit, and the induced electric field (in 3-direction) can be expressed as

(4) $$E_{3}=\frac{d_{33}^{e}F_{0}}{\pi r_{p}^{2}\varepsilon_{0}\left(\varepsilon_{33}^{T}-1\right)}=\frac{d_{33}^{e}B_{l}J_{0}}{\varepsilon_{e}\pi r_{p}^{2}}\;$$

where $d_{33}^{e}$ is the effective piezoelectric coefficient of the drum transducer, which is dependent on the geometry of the drum transducer [22], *F*_0_ is the amplitude of *F_r_*, *r_p_* is the radius of the piezoelectric disk, ε_0_ is the permittivity of vacuum, ε_0_ = 8.85 × 10^−^^12^ F/m, and $\varepsilon_{33}^{T}$ is the relative permittivity. The induced voltage in the application of *F_r_* is

(5) $$V_{3}=E_{3}t_{p}=\frac{d_{33}^{e}B_{l}J_{0}t_{p}}{\varepsilon_{e}\pi r_{p}^{2}}\;$$

The sensitivity of the piezoelectric heterostructure *S* is obtained as

(6) $$S=\frac{V_{3}}{I_{0}}=\frac{d_{33}^{e}B_{l}t_{p}}{\varepsilon_{e}{\left(\pi r_{p}r_{c}\right)}^{2}}\;$$

It is clear that the sensitivity *S* is proportional to $d_{33}^{e}$. The use of the piezoelectric drum transducer with a high effective piezoelectric coefficient can potentially improve the sensitivity of the proposed device. For energy harvesting applications, the output power across the external resistive load *R_L_* can be expressed as

(7) $$P=\frac{V_{L}^{2}}{R_{L}}=\frac{{\left(\omega CV_{3}\right)}^{2}R_{L}}{2[1+\left(\omega CR_{L}{)}^{2}\right]}\;$$

where *R_L_* is the load voltage and *C* is the capacitance of the piezoelectric disks. The impedance of the piezoelectric drum transducer *R_S_* depends on the angular frequency *ω* and the capacitance *C*, and *R_S_* = 1/(*ωC*) [23]. The maximal power can be found at *R_L_* = *R_S_*, which is given by

(8) $$P_{max}=\frac{\pi fC}{2}{\left(\frac{d_{33}^{e}B_{l}J_{0}t_{p}}{\varepsilon_{e}\pi r_{p}^{2}}\right)}^{2}\;$$

It can be seen from Equation (8) that *P_max_* is proportional to the square of *B_l_*. *P_max_* can be improved using the magnetic circuit shown in Figure 1, which improves *B_l_* due to the specific arrangement of the magnets.

## 3. Results and Discussion

A prototype was fabricated according to Figure 1 to study the feasibility of the proposed piezoelectric heterostructure. The material of the piezoelectric disks is PZT5H. The radius and the thickness of each piezoelectric disk are 7.5 mm and 0.2 mm, respectively. The brass disk (with a radius of 10mm and a thickness of 0.2 mm) and the piezoelectric disk are bonded using insulate epoxy adhesive, and are cured under load.

**Figure 3:**
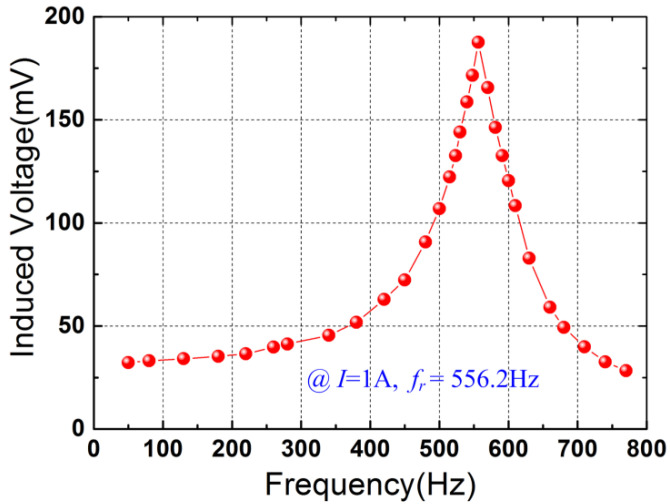
Induced voltage as a function of the current frequency at 1 A.

The frequency-voltage response of the prototype was investigated. Figure 3 shows the induced voltage versus the frequency at 1 A. It can be seen from Figure 3 that the induced voltage attains 187.6 mV at the resonant frequency of *f_r_* = 556.2 Hz in the given frequency range (50 Hz–770 Hz). The 3 dB bandwidth Δ*f* of the device is 67.4 Hz. As the sensor operates at the power frequency (50 Hz or 60 Hz), the broad 3 dB bandwidth might be beneficial for the improvement of the sensitivity.

**Figure 4:**
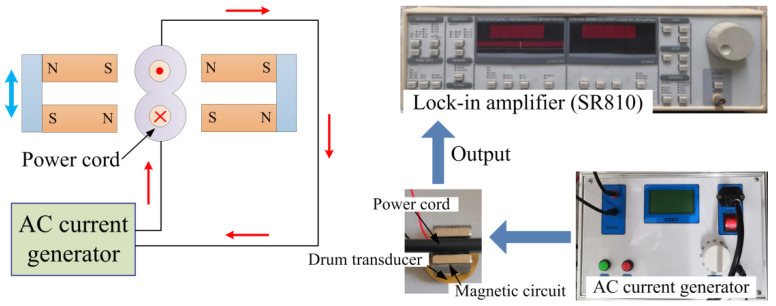
Experimental set-up for 50 Hz current sensing experiments.

**Figure 5:**
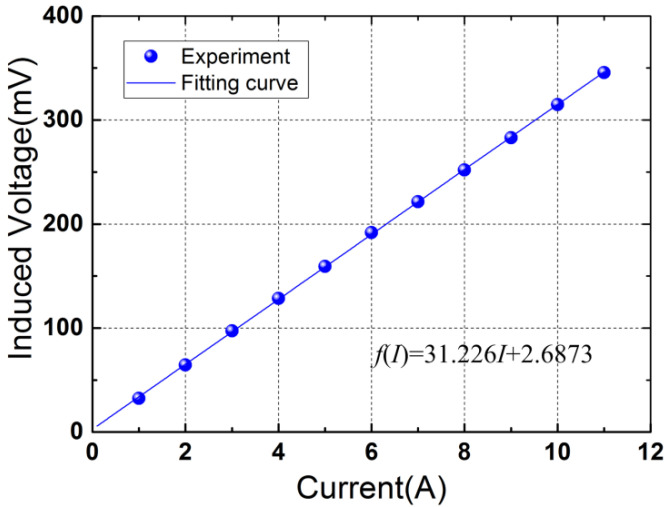
Induced voltage as a function of the current at the power frequency of 50 Hz.

Figure 4 shows the experimental set-up of the current sensing experiments. A current generator is used to generate electric currents in the power cord. The power cord passes through the magnetic circuit. The magnetic circuit experiences an enhanced force when the power cord is energized, which induces an output voltage of the piezoelectric drum transducer. The output voltages of the sensor are monitored using a lock-in amplifier. Figure 5 plots the induced voltage versus the applied electric current at the power frequency of 50 Hz. A good linear response is observed in Figure 5. The voltage varies from 32.29 mV to 345.68 mV when the electric current increases from 1 A to 11 A. The average sensitivity reaches 31.34 mV/A due to the high effective piezoelectric coefficient of the drum transducer. The fitting curve is obtained using the least squares method (with a slope of 31.226 and an intercept of 2.6873), which is given by

(9) f(I)=31.226I+2.6873 

The corresponding correlation coefficient is 0.99995853. The linearity is analyzed based on the fitting curve and the experimental data, which is calculated by $\delta =\left(\Delta V_{max}/Y\right)\times 100\%$. Here, Δ*V_max_* denotes the maximum deviation, and Y is the full scale output. Using the data in Figure 5, a high linearity of 0.49% is obtained, which is very favorable to 50Hz or 60Hz current sensing in electric power grids.

**Figure 6:**
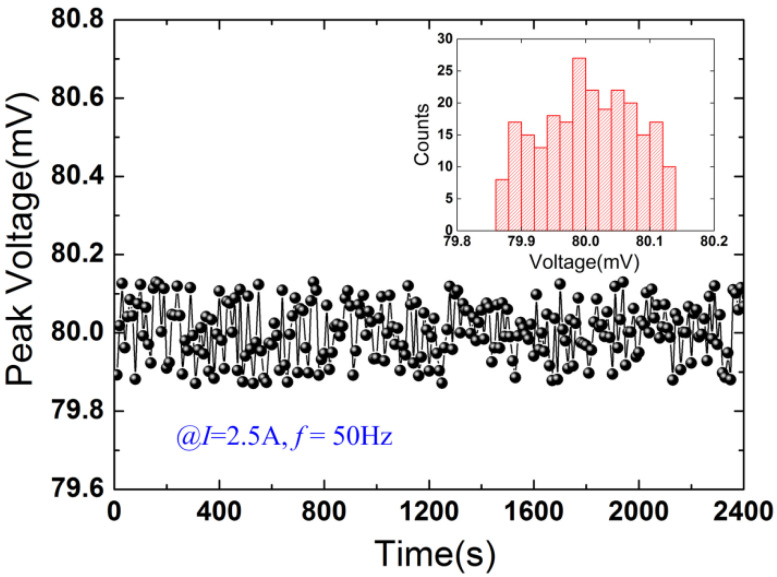
Induced voltage as a function of time at 2.5 A. The inset plots the histogram of the voltages.

Figure 6 plots the induced voltage versus time at 2.5 A (the number of the measured voltages is 240). The inset of Figure 6 shows the histogram of the induced voltages. The standard deviation is calculated by

(10) $$\sigma =\sqrt{\frac{\sum_{i=1}^{n}\nu_{i}^{2}}{n-1}}\;$$

where $\nu_{i}$ is residual error, $\nu_{i}=V_{i}-\overset{-}{V}$, and $\bar{V}$ is the average value of the voltages. Using the sample points (*n* = 240), $\bar{V}$ and *σ* are calculated to be 80.0038 mV and 0.07224 mV, respectively. Normal distribution can be utilized to analyze the sample points based on the histogram. The confidence interval with a confidence level of 99.99% can be expressed as

(11) $$S=\left[\overset{-}{V}-\varepsilon ,\overset{-}{V}+\varepsilon \right]\;$$

where $\varepsilon =4\sigma /\sqrt{n}$. The uncertainty of the sensor is given by

(12) ξ=±ε 

According to the measured voltages shown in Figure 6, the confidence interval is obtained to be [79.98515 mV, 80.02245 mV] for a 99.99% confidence level, and the uncertainty of the sensor reaches 0.01865 mV.

**Figure 7:**
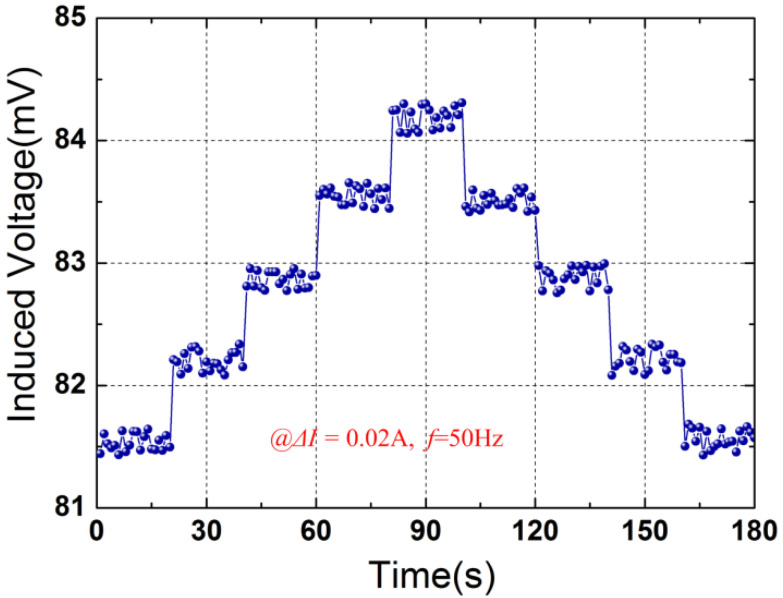
Output voltage as a function of time under small current step changes.

Figure 7 shows the resolution of the current sensor to small current variation (Δ*I =* 0.02 A) at the frequency of 50 Hz. From Figure 7, an obvious step-change of the induced voltage was observed by adjusting the amplitude of the current within 180 s. A current change as small as 0.02 A is clearly distinguished. It is estimated that this resolution could be further improved by adopting the magnets with a higher remnant flux density and the piezoelectric material with higher piezoelectric coefficient (e.g., PMN-PT). The proposed sensor possesses the characteristics of high sensitivity, high linearity, high resolution, and low costs. Furthermore, the sensor operates without the requirement to wholly encircle the power cord. These features facilitate the current sensing of the proposed device in realistic applications.

**Figure 8:**
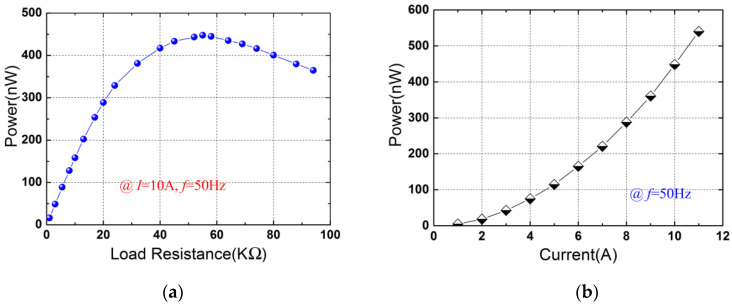
Output powers for energy harvesting application: (**a**) Output power as a function of load resistance for *I* = 10 A; (**b**) Maximal power versus electric current at the frequency of 50 Hz.

The proposed device can be used to harvest magnetic field energy from the power cord. A resistance box was connected to the output of the device. By changing the load resistance, the load voltage increases with the load resistance. The experimental powers were obtained based on the measured load voltages and the corresponding load resistances. Figure 8a plots the experimental output power as a function of the load resistance at the current of 10 A (*f* = 50 Hz). It can be seen from Figure 8a that the power does not always increase with the resistance. The power reaches a maximum value of 447.9 nW across the resistance of 55 KΩ. Figure 8b shows maximal power versus the electric current at the frequency of 50 Hz. The maximal powers are calculated according to the optimal load resistances and the corresponding load voltages. It can be seen from Figure 8b that the power exhibits an approximate quadratic increase. The power increases from 4.65 nW to 539.8 nW when the current is increased from 1 A to 11 A. It should be noted that the electrical field energy harvested by the device is ignored as the proposed piezoelectric heterostructure is intended for low-voltage applications (e.g., 220 V).

## 4. Conclusions

In this paper, a self-powered electric current sensor based on a piezoelectric drum transducer is presented. The superposition of the Ampere forces on the conductors of the power cord enhances the response of the sensor to the electric current, which improves the sensitivity of the device. Some conclusions to this study are summarized as follows. (1) The induced voltage of the sensor has a highly linear relationship with the input current, and a high linearity of 0.49% is obtained with an average sensitivity of 31.34 mV/A. (2) The resolution of the sensor was experimental investigated and a high resolution of 0.02 A is attained at the power frequency of 50 Hz, indicating that a step current change as small as 0.02 A can be distinguished by the proposed sensor. (3) The feasibility of the device for harvesting magnetic field energy from two-wire power cords was verified. The prototype generates a load power of 447.9 nW with a matching load resistance of 55 KΩ at a current of 10 A.

## Data Availability

The data used to support the findings of this study are included within the article.
